# Evaluation of Dental Fear in Children during Dental Visit using Children's Fear Survey Schedule-Dental Subscale

**DOI:** 10.5005/jp-journals-10005-1178

**Published:** 2013-04-26

**Authors:** Sunil Raj, Manisha Agarwal, Kiran Aradhya, Sapna Konde, V Nagakishore

**Affiliations:** Professor, Department of Pedodontics, AECS Maaruti Dental College Bengaluru, Karnataka, India; Department of Pedodontics, AECS Maaruti Dental College Bengaluru, Karnataka, India; Department of Pedodontics, AECS Maaruti Dental College Bengaluru, Karnataka, India; Department of Pedodontics, AECS Maaruti Dental College Bengaluru, Karnataka, India; Department of Pedodontics, AECS Maaruti Dental College Bengaluru, Karnataka, India

**Keywords:** Dental fear, Dental anxiety, Survey Schedule

## Abstract

Fear of dental treatment in children has been recognized as a source of serious health problems and it may persist into adolescence, which may lead to a disruptive behavior, during dental treatment. In order to prevent this psychometric method namely the dental subscale of the children's fear survey schedule (CFSS-DS) is a well-known psychometric scale that was developed by Cuthbert and Melamed in 1982 for assessing dental fear in children. The present study was to evaluate dental fear in children during first dental visit using CFSS-DS between three different age group 4 and 6 years, 7 and 9 years, 10 and 14 years children to select fearful and nonfearful children from a larger reference population and to estimate the dental fear children. Total 600 children show CFSS-DS of 27.17 ± 5.3385, 307 were girls (51.17%) and they showed CFSS-DS of 27.50 ± 5.060 and 293 were boys (48.83%) and they show CFSS-DS 26.84 ± 5.617. This shows that there were no significant difference in fear between boys and girls. In 4 to 6 years show total CFSS-DS 28.78 ± 5.742, 7 to 9 years show that mean and standard deviation of CFSS-DS 27.81 ± 4.783, 10 to 14 years show that mean and standard deviation of CFSS-DS 25.93 ± 5.586. Fear scores were highest for ‘injections', ‘choking', ‘noise of dentist drilling', ‘dentist drilling which was not significant between boy's and girl's but item, ‘having somebody look at you’ showed that significant differences in fear scores between boys and girls in present study. The present study concluded that dental fear decreased as age increased. Total fear scores also exhibited no strong overall sex difference or age by sex interaction.

d> Raj S, Agarwal M, Aradhya K, Konde S, Nagakishore V. Evaluation of Dental Fear in Children during Dental Visit using Children's Fear Survey Schedule-Dental Subscale. Int J Clin Pediatr Dent 2013;6(1):12-15.

## INTRODUCTION

Extreme dental fear is a universal problem. It leads to the avoidance of dental treatment and the adverse consequences to the patient's oral and psychological health. Dental fear is defined as a specific anxiety, which is the predisposition for a negative experience in the dental surgery.^1^ Dental fear may cause frequent and serious problem for both patient and dentist. The etiology of dental fear in children is multifactorial. Dental fear has been related to personality, increased general fears, previous painful dental experiences, parental dental fear, age and gender. Girls and younger children are most often reported as more fearful than boys and older children.

For this purpose different types of measuring scales have been used to assess the dental fear. Four types of dental fear assessing scales in children are: Rating behavior during dental visits (e.g. the Frankl Behavior Rating Scale; FBRS), physiological measures (e.g. pulse rate, basal skin response and muscle tension), projective techniques [e.g. Facial Image Scale (FIS) or Children's Dental Fear Picture Test (CDFP)] and psychometric scales.^2^

Among them psychometric method namely the dental subscale of the children's fear survey schedule (CFSS-DS) is a well known psychometric scale that was developed by Cuthbert and Melamed in 1982 for assessing dental fear in children. It has been shown to have good reliability validity, and recently has been used in several countries and translated into several languages. CFSS-DS has been shown to be better in some situations than other scales, such as the Venham Picture Test and the Dental Anxiety Scale.^3^

Studies of children and adults have shown that dental fear is associated with less favorable self-care behavior, avoidance of dental care and also with poorer health outcomes. Nevertheless, some studies have shown that there was no correlation between dental fear and caries.^4^

Aim of the present study is to evaluate dental fear in children in different age group between 4 and 6 years, 7 and 9 years, and 10 and 14 years during First dental visit using CFSS-DS.

## SUBJECTS AND METHODS

The sample comprised of 600 children who were 4 to 14 years old both male and female visiting the Department of Pedodontia and Preventive Dentistry at AECS Maaruti College of Dental Sciences and Research Center, Bengaluru, for a period of 18 months (1.5 year). The study population was of low socioeconomic status. Children for the study were randomly selected with good general health who were cooperative during the study and children who had no previous dental treatment and children with disabilities were excluded from the study. The study was approved by ethical clearance committee from the institution AECS Maaruti College of Dental Sciences and Research Center.

CFSS-DS developed by Cuthbert and Melamed in 1982 and has been shown to be reliable and valid. The CFSS-DS consists of 15 items (Table 1) covering different aspects of the dental situation was used in the study. All Items were filled by dentist during first visit to the dental hospital.

**Table 1:** d> Children's fear survey schedule—dental subscale

*S.no.*	*Items*
1.	Dentists
2.	Doctors
3.	Injections
4.	Having somebody examine your mouth
5.	Having to open your mouth
6.	Having a stranger touch you
7.	Having somebody look at you
8.	The dentist drilling
9.	The sight of the dentist drilling
10.	The noise of the dentist drilling
11.	Having somebody put instruments in your mouth
12.	Choking
13.	Having to go to the hospital
14.	People in white uniform
15.	Having the dentist clean your teeth

**Graph 1:**
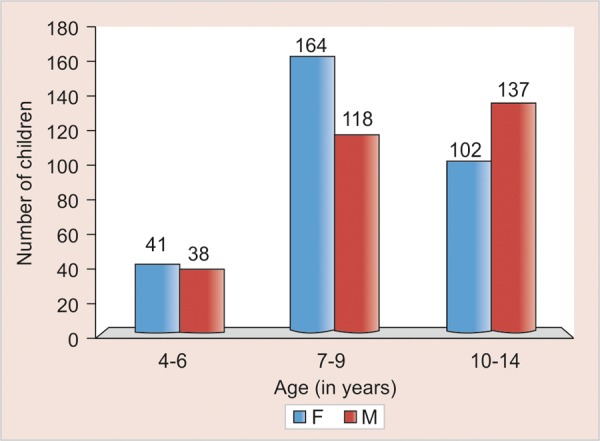
Representation of the study population according to age and sex

All values are tabulated and the mean CFSS-DS scores were calculated for the study population and also for girls and boys of all different age. Analysis of variance was performed to test the difference in fear scores between different age group 4 and 6 years, 7 and 9 years, 10 and 14 years between boys and girls (Graph 1).

## RESULT

In the Table 2, 600 children were examined in the age group of 4 to 14 years with mean and standard deviation of CFSS-DS 27.17 ± 5.3385, 307 were girls (51.17%) and showed the mean and standard deviation of CFSS-DS 27.50 ± 5.060, 293 were boys (48.83%) and showed the mean and standard deviation of CFSS-DS 26.84 ± 5.617. This shows that there were no significant difference in fear between boys and girls.

In Table 3, age group of 4 to 6 years with 80 children show the mean and standard deviation of CFSS-DS 28.78 ± 5.742 out of which 59 where nonfearful with mean and standard deviation of CFSS-DS 26.20 ± 3.263,17 were moderately fearful with mean and standard deviation CFSS-DS 34.38 ± 2.125 and 4 were fearful with mean and standard deviation of CFSS-DS 44.5 ± 1.291, of which there is not significant difference between the fear in 4 and 6 years of age group. In the age of 7 and 9 years with 280 children show that mean and standard deviation of CFSS-DS is 27.81 ± 4.783 and out of which 226 nonfearful show that mean and standard deviation is 26.12 ± 3.400, whereas 47 moderate fearful show that mean and standard deviation of CFSS-DS is 33.94 ± 1.621, and seven are fearful with mean and standard deviation of CFSS-DS 41.00 ± 2.449, where fearful of which is not significant difference between the fear in 7 and 9 years of age group. In the age group of 10 and 14 years with 240 children in the age show that mean and standard deviation of CFSS-DS is 25.93 ± 5.586, where 209 are nonfearful and show the mean and standard deviation of 24.52 ± 4.344, whereas 27 are moderately fearful and show the mean and standard deviation of 34.30 ± 1.728, whereas four are fearful and show the mean and standard deviation of 42.75 ± 2.630, of which is not significant difference between the fear in 10 and 14 years of age group.

**Table 2:** **Table 2:** Mean and standard deviation of CFSS-DS for total population and boys and girls

*Age (in years)*		*Girls*				*Boys*				*Total*		
		*N*	%	*Mean ± SD*		*N*	%	*Mean ± SD*		*n*	%	*Mean ± SD*
Total		307	51.17	27.50 ± 5.060		293	48.83	26.84 ± 5.617		600	100.00	27.17 ± 5.3385

p = 0.0026 (<0.05)

**Table 3:** **Table 3:** Mean scores and standard deviation of nonfearful, moderate and fearful according to age group

*Age (in years)*		*Nonfearful (<32)*			*Moderate (32-39)*			*Fearful (>39)*			*Total*	
		*N*	*Mean ± SD*		*N*	*Mean ± SD*		*n*	*Mean ± SD*		*n*	*Mean ± SD*
4 to 6		59	26.20 ± 3.263		17	34.38 ± 2.125		4	44.5 ± 1.291		80	28.78 ± 5.742
7 to 9		226	26.12 ± 3.400		47	33.94 ± 1.621		7	41.00 ± 2.449		280	27.81 ± 4.783
10 to 14		209	24.52 ± 4.344		27	34.30 ± 1.728		4	42.75 ± 2.630		240	25.93 ± 5.586
Total		494	25.46 ± 3.890		91	34.12 ± 1.741		15	42.50 ± 5.348		600	27.18 ± 5.348

p = 0.081 (>0.05)

**Table 4:** **Table 4:** Mean and standard deviation of degrees of affraidness

*S.no.*	*Items*	*Girls (n = 307)*		*Boys (n = 293)*		*All (n = 600)*		*p-value*
		*Mean ± SD*		*Mean ± SD*		*Mean ± SD*		
1.	Dentists	1.156 ± 0.373		1.129 ± 0.356		1.143 ± 0.364		0.3645
2.	Doctors	1.153 ± 0.369		1.119 ± 0.345		1.136 ± 0.358		0.2447
3.	Injections	3.785 ± 1.128		3.689 ± 1.203		3.738 ± 1.165		0.3135
4.	Having somebody examine your mouth	1.228 ± 0.599		1.150 ± 0.443		1.190 ± 0.530		0.0713
5.	aving to open your mouth	1.176 ± 0.480		1.129 ± 0.472		1.153 ± 0.476		0.9869
6.	Having a stranger touch you	1.374 ± 0.749		1.375 ± 0.861		1.373 ± 0.805		0.9879
7.	Having somebody look at you	1.195 ± 0.583*		1.092 ± 0.390*		1.145 ± 0.501		0.0116*
8.	The dentist drilling	2.306 ± 0.769		2.252 ± 0.871		2.280 ± 0.820		0.4206
9.	The sight of the dentist drilling	2.573 ± 0.761		2.573 ± 0.843		2.573 ± 0.801		0.9999
10.	The noise of the dentist drilling	3.107 ± 1.028		3.102 ± 1.099		3.105 ± 1.062		0.954
11.	Having put instrument in mouth	1.866 ± 0.770		1.832 ± 0.829		1.850 ± 0.799		0.6027
12.	Choking	2.596 ± 0.732		2.597 ± 0.786		2.596 ± 0.758		0.9871
13.	Having to go to the hospital	1.225 ± 0.476		1.184 ± 0.430		1.205 ± 0.454		0.2753
14.	People in white uniform	1.208 ± 0.500		1.157 ± 0.417		1.183 ± 0.462		0.1764
15.	Having the dentist clean your teeth	1.469 ± 0.682		1.457 ± 0.684		1.463 ± 0.682		0.8297
Total	1.828 ± 1.075	1.789 ± 1.091		1.809 ± 1.083				

**Graph 2:**
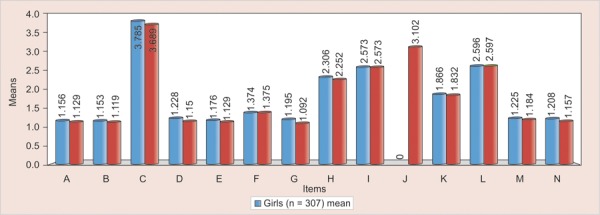
Representation of means of items according to sex

The Table 4 shows that mean and standard deviation for 15 Item are no significantly different between boys and girls except for item 7 (having somebody look at you) shows which the significant difference in the mean score between boys and girls. The most feared items for both boys and girls were item 3 (injections) (3.738 ± 1.165), item 10 (noise of the dentist drilling) (3.105 ± 1.062), item 12 (choking) (2.596 ± 0.758), Item 9 (sight of dentist drilling) (2.573 ± 0.801), Item 8 (dentist drilling) (2.280 ± 0.820) with the least fearful item 2 (1.136 ± 0.358) (doctors) (Graph 2).

## DISCUSSION

Fear of dental treatment in children has been recognized as a source of serious health problems and it may persist into adolescence, which may lead to a disruptive behavior, during dental treatment. In order to prevent this type of disruptive behavior, preferably by means of appropriate pediatric management techniques, it is imperative to identity the dentally anxious child at the earliest possible age.^1^ The aim of this present study was to evaluate the level of dental fear by using CFSS-DS which was given by Cuthbert and Melamed in 1982 among 4 to 14 years old children who visited to the Department of Pedodontia and Preventive Dentistry at AECS Maaruti College of Dental Sciences and Research Center, Bengaluru, for dental treatment. By using the cutoff value of >32, the mean dental score of CFSS-DS in the study was 27.17 ± 5.3385.

The CFSS-DS scores in the present study were similar to the data of previous study in Turky ( 28.1)^9^, and USA (28.1)^4^ children. The mean score was higher than the finding in Finlands (22.1),^7^ Sweden (23.1)^8^ and the Netherland (23.2)^13^ and lower than the finding in Singapore (30.6)^6^Canada (31.9)^12^ and China (35.7).^14^ The present study also showed that there is no significant difference between boys and girls which is similar to previous studies. But same study had shown that girl's score are higher on the CFSS-DS.^5,6,13^ The data obtained in the present study showed that 17.5% of study population suffer from some degree of dental fear possibly increasing with dental treatment. Two cutoff points have been set representing different degree of dental fear in children. The CFSS-DS scores of 39 and above (2.5%) were found to represent high dental fear in children. Likely to interfere with dental treatment, a borderline area for dental fear was set at score between 32 and 39^10,14^ with children scoring in this range (15%) also suffer some degree of dental fear and may be at risk for developing high dental fear or phobia. This group of children are of special interest in the study of dental fear. Since by providing extra attention and guidance for these children, the development of high dental fear may be prevented.

No significant difference in fear scores between boys and girls were found in the present study and it is also found that dental fear decrease with increasing age. This result is partly due to the fact that this relation may not be a linear one and that it may be affected by other aspects. In the present study, children were most afraid of injections, noise of the dentist drilling, choking, sight of dentist drilling, dentist drilling which is confirmed from the previous study.^11,12^ With providing the extra attention during the dental treatment for the children, we can reduce fear of dentist and dental treatment at an early age and we can provide a good dental treatment.

Dental anxiety is a serious problem which negatively affects the oral health of children and adults. Early detection of the causes of fear is very important in the solution of the problem. It is recognized that children who witness fear in their parents are likely to acquire that outlook and as a result experience painful experience at an early age and that this is an important factor related to the problem. To reduce the level of dental fear among children, attention needs to be paid to the use of epidemiologic concepts of clinical risk assessment using caries activity tests and early intensive preventive effects, such as fissure sealants, routine oral health examination, oral hygiene instruction and parental education to prevent the child from experiencing pain and reduce the need for injections and extensive dental treatment at very early age.

## CONCLUSION

The results of the present study showed that dental fear in 4 to 14-year-old decreased as age increased. Total fear scores also exhibited no strong overall sex difference or age by sex interaction.
